# Attitudes toward sexual and reproductive health among adolescents and young people in urban and rural DR Congo

**DOI:** 10.1186/s12978-018-0517-4

**Published:** 2018-05-04

**Authors:** Fidèle Mbadu Muanda, Ndongo Parfait Gahungu, Francine Wood, Jane T. Bertrand

**Affiliations:** 1 Programme National de Santé de la Reproduction (National Program for Reproductive Health) and Programme National de Santé de l’Adolescent (National Program for Adolescent Health), Ministry of Health, Avenue des Cliniques n 43/cliniques kinoises, Commune de la Gombe, Kinshasa Democratic Republic of Congo; 2 L’Institut Supérieur de Dévéloppement Rural, Avenue des Cliniques n 43/cliniques kinoises, Commune de la Gombe, Kinshasa Democratic Republic of Congo; 3 Department of Global Community Health and Behavioral Sciences, Tulane University School Public Health and Tropical Medicine, 1440 Canal Street, New Orleans, LA 70112 USA; 4 Department of Global Health Management and Policy, Tulane University School Public Health and Tropical Medicine, 1440 Canal Street, New Orleans, LA 70112 USA

**Keywords:** Contraception - sexual and reproductive health, Adolescents - young people - Democratic Republic of Congo (DRC), Qualitative research, Focus group discussion

## Abstract

**Background:**

In the Democratic Republic of Congo (DRC), onset of sexual intercourse is initiated during adolescence, however only two in ten sexually active unmarried women are using modern contraception. Improving adolescents’ and young peoples’ knowledge and practices related to sexual and reproductive health (SRH) is necessary to improve health outcomes. However, little is known about the SRH attitudes and needs among young people in the DRC. The study aims to contribute to the available evidence by examining adolescents’ and young people’s insights on their cultural norms, practices and attitudes towards SRH services.

**Methods:**

Fourteen focus group discussions were conducted with a total of 224 adolescents and young people aged 15–24 years in urban and rural areas of the DRC. The topics discussed and age groups of participants differed somewhat in the urban and rural areas. Data were analyzed to identify themes in the participants’ discussion of their attitudes towards SRH.

**Results:**

Regardless of age differences, common themes emerged. Both in rural and urban areas premarital sex was largely sanctioned by peers but not adults; adolescents feared pregnancy and had limited knowledge of contraceptive methods. Many were misinformed that certain common pharmaceutical products (e.g., decaris) prevent pregnancy. Key barriers to accessing contraception from health facilities and pharmacies included shame and stigma; urban participants also cited cost and judgmental attitudes of health providers.

**Conclusion:**

Addressing the SRH needs of adolescents and young people can have life-long protective benefits. Increasingly decision-makers and gatekeepers in the DRC are accepting the concept of providing SRH services and information to young people. This study shows the pressing need for information and services for young people in both urban and rural areas. The continued expansion SRH programming to all health zones and the developed of the National Strategic Plan for Health and Wellbeing of Adolescents and Youth 2016–2020 are steps toward that goal.

## Plain English summary

Evidence suggests adolescents in developing countries face a range of challenges when they choose to access sexual and reproductive health services. We conducted a study in selected health zones in urban and rural areas in the Democratic Republic of Congo to explore cultural norms, attitudes, and practices of adolescents and young people regarding sexual and reproductive health (SRH) services. We held focus group discussions in selected health zones with young people aged 15–19 years in the urban areas and 18–24 years in rural areas. We found that young people in urban and rural areas have very similar attitudes towards SRH services, practices and cultural norms. Young people were accepting of premarital sex, feared pregnancy, had limited knowledge of contraceptive methods, erroneously used common pharmaceutical products to prevent pregnancy, experienced barriers when purchasing contraception from health facilities and pharmacies; and felt shame and stigma. Urban adolescents (only) experienced cost-related barriers; and only urban young women more frequently cited judgmental attitudes of providers. In the rural areas, adolescents felt that using contraception was the responsibility of the woman. In order to improve the use of services, these norms, practices and attitudes of the young people need to be taken into account and addressed in the design and delivery of programs to young people and adolescents.

## Background

The Democratic Republic of Congo (DRC), with a population of 79.9 million, has the third highest total fertility rate (TFR) in the world; 6.5 children, varying from 5.4 (urban) to 7.3 (rural) [1]. By 2050, it will be the 8th most populous country in the world [2]. It is among the poorest countries of the world ranking 178 out of 188 countries for the Human Development Index [3].

Similar to other high fertility countries, the population of the country is young; 52% are under 15 years [1]. Early sexual experience and early childbearing contribute to maintaining high levels of fertility. According to the 2013–14 Demographic and Health Survey (DHS), 27% of women aged 15–19 have begun childbearing: 21% are mothers and 6% are currently pregnant [1]. Half of women initiate sexual intercourse by age 16.8; men on average at 17.6 years [1]. Modern contraceptive use is low: only two in ten sexually active unmarried women are using modern contraception, with the male condom being the most popular method [1]. Prior research indicates that adolescent women experience greater unmet need of contraception than their adult counterparts [4, 5].

Globally, the DRC ranks 8th in the top ten countries with the greatest number of women aged 20–24 who gave birth by age 18 [6]. Even within the region, the adolescent fertility rate (124 per 100 women aged 15–19 years) for the DRC is high in comparison to that of sub-Saharan Africa (109 per 1000) [7]. Consistent with these findings, modern contraceptive use among women 15–24 in the DRC (15.5%) is among the lowest in sub-Saharan Africa [1, 5].

Adolescent pregnancy and childbearing both threaten the health of the young woman and limit her future opportunities for education and employment. Research from multiple countries shows that adolescent pregnancy can be detrimental to the health of the mother and newborn because of the higher risk of maternal complications including postpartum hemorrhaging, eclampsia, preterm delivery and systemic infections [4, 8, 9]. Newborns have a higher chance of suffering low birth weight and early neonatal death. In addition, pregnancies during adolescence can have negative social and economic effects on families and communities [10, 11]. Despite the high proportion of adolescents and youth between ages 15–24 years, little research has been conducted on sexual and reproductive health (SRH) among this group in the DRC [12, 13]. Rather, much of the evidence on young people’s contraception use and preferences for reproductive health services have been conducted in other countries [14–17].

Multiple factors contribute to high fertility norms in the DRC, including high adolescent fertility. Romaniuk points to social, cultural and economic factors: low levels of education among the population, especially girls; the low status of women, who become legally subordinate to their husbands at the time of marriage; few alternative economic opportunities; strong cultural norms that encourage large families; expectations from the husband’s family for numerous children in return for the dowry paid for the woman, among others [18]. The DRC is a predominantly Christian country, with a large percentage belonging to the Catholic Church [1]. Although the dictates of the Catholic Church against “artificial contraception” are sometimes cited, the Catholic Church is not a major barrier to contraceptive service use [19].

In the past 5 years the number of programmatic initiatives to address adolescent and young people’s sexual and reproductive health has steadily increased in the DRC, largely in urban areas (e.g., Kinshasa, the capital city) [20]. Whereas the DHS surveys provide valuable quantitative data on pregnancy rates, age at first sex, percentage using modern contraception, and related indicators, the emerging programmatic efforts will benefit from the rich, insightful findings that characterize qualitative research. To date, there is a dearth of qualitative research in the published literature on attitudes and behaviors among adolescents and young people in the DRC. The current article is intended as a means of building this literature.

The objective of this analysis is to present, compare and contrast the attitudes and perceptions of adolescents and young people in an urban and a rural context of the DRC on issues related to marriage, pre-marital sex, pregnancy prevention and contraceptive use.

## Methodology

The data for this analysis came from two different studies - one conducted in the capital city of Kinshasa (urban), the other in three rural health zones participating in the ASSP (*Accès au Soins de Santé Primaire*) Project. A single individual (first author on this paper) served as study director for both, thus bringing consistency in the methodological approach to data collection and analysis. The objective of the primary study was to collect qualitative data from both urban and rural sites on barriers to contraceptive use among adult women and men in these communities, the results of which have been published elsewhere [12, 13]. We took advantage of having the research teams in the field (especially in the remote rural areas) to also explore SRH attitudes among young women and men in these same communities.

Although the age groups and topics discussed were not identical, the authors considered it valuable to compare and contrast the views of adolescents and youth from these very different settings on SRH issues.

### Study setting and population

In Kinshasa, we purposely selected two health zones that were centrally located, densely populated and had better than average access to contraceptive services (to reduce the influence that lack of access could play on the non-use of contraceptive methods): Kalamu II and Bumbu.

In each of three rural provinces – Maniema, Kasai, North Ubangi – we selected one “strong” and one “weak” health zone, based on a widely used measure of output in family planning programs, couple-years protection (CYP) [21]:

1. Maniema: Alunguli (strong) and Kampene (weak)
2. Kasai: Mutoto (strong) and Mweka (weak)
3. North Ubangi: Bili (strong) and Bosobolo (weak)

We were interested in comparing the attitudes of young people from urban and rural areas, given the differential fertility of the two. For example, the adolescent birth rate in Kinshasa as of 2013–14 was 67 per 1000 women 15–19 years old, compared to Kasai (148 per 1000), Maniema (161 per 1000), and Nord Ubangi (166 per 1000) [1].

### Recruitment

In Kinshasa, the data collection team worked with nurses to invite a convenience sample of adolescents to participate in individual and confidential interviews to determine their eligibility. Invitations to participate in the focus group discussions (FGD) were extended to adolescents who fulfilled the eligibility criteria: to be 15–19 years old, unmarried, and sexually active (have had sexual intercourse within the last 3 months). In the rural area the research team with the assistance of community leaders recruited youth from the community who met the following eligibility criteria: 18–24 years old, unmarried, with no children.

### FGD guide

The framework of Sexual and Reproductive Health Lifestages provides a theoretical basic for the topics covered; the phases included pre-sexual, sexual & pre-reproductive, and sexual & reproductive [22]. Each question from the guide would allow for participants to directly answer the question or make related comments not necessarily on the guide (e.g., provider attitudes toward adolescents who seek contraceptive services).

Because the research team anticipated that adolescents and young people living in urban areas would have greater exposure to issues related to contraception than those living in remote rural areas, the urban FGD guide focused on: knowledge of contraceptive methods, teenage pregnancy; obstacles and barriers to contraceptive use; attitudes to teenage pregnancy and the use of contraceptive methods; and the social acceptability of contraceptive use. The rural FGD guide included a series of topics that would provide greater context to sexual behavior and need for contraception, such as the desire for a family, preferred family size, attitudes toward premarital sex, and adolescent’s motivation to engage in premarital sex; the guide also touched on knowledge of modern methods, and perceptions of provider attitudes towards adolescents’ use of contraceptives.

### Data collection

The data collection team for each FGD consisted of a moderator and a note-taker who were experienced in FGD. Prior to the data collection, they participated in a refresher training on FGD methodology, the study protocol, questions to cover, translation of specific questions into the local language, and pretesting of the guides.

The urban FGD were conducted in February 2015, the rural FGD in August 2015. In Kinshasa, we conducted eight FGD among males and females aged 15–19 years old with 10 participants per group. In the rural area four FGD were organized in each of 6 health zones, with 12 participants each. The focus group method allowed all, regardless of their ability to read or write, to participate. In both the urban and rural areas, the groups were composed of members of similar age, sex, and marital status. The homogeneity of each group created an atmosphere where participants were comfortable and readily shared their perspectives.

### Data analysis

The sessions were conducted in the local language and audiotaped. The transcribers listened to the tapes in the local language and then transcribed them into French. Subsequently the research team coded the participants’ responses by theme and sub-theme, to facilitate thematic content analysis. Once the data were coded, the team developed a matrix that captured the key ideas by theme, location, and gender. In addition, they identified verbatim quotes that succinctly captured common responses for use in the article. In this paper, we present key findings for urban and rural groups separately, indicating the gender of the respondents. Unpublished reports of these findings are available in French [21, 23].

### Ethical approval

The study received IRB approval from Tulane University [urban (14-669918); rural (717117)] and the University of Kinshasa School of Public Health [urban (ESP/CE/057/2014); rural (ESP/CE/0901/2015)].

Prior to data collection, the moderator clarified study objectives and ensured confidentiality for participants. Each participant in the urban and rural health zones signed a consent form, prior to participation in the FGD.

## Results

### Characteristics of study participants

In total, we recruited 224 participants: 112 females and 112 males. In Kinshasa, there were a total of 80 participants aged 15–19 years: 40 males aged and 40 females. All participants were unmarried and majority (90%) had some secondary school education. Their median age was 18 years and majority reported not having children (94%).

In the rural area, 144 participants aged 18–24 years were recruited: 72 females and 72 males. We did not collect data on the socio demographic characteristics of the rural participants. However, the data from the 2013–2014 DHS show that 43% of young people aged 15–24 years from rural areas of these provinces have some secondary or higher education and 63% are employed [1]. The DHS also shows that Kinshasa has the highest percentage of young people with secondary or higher education (89.9%) and North Ubangi has the lowest (43.4%) [1]. The DRC as a country is predominantly Christian, including Catholics, Protestants, Evangelicals, and Kimbanguists [1].

### Urban population

Both males and females aged 15–19 from Kinshasa were very open and forthcoming with their attitudes on multiple issues related to pregnancy and contraceptive use.

#### Attitudes towards pregnancy

Pregnancy was a recurrent theme in the FGD with the urban population. Overall, adolescent girls and boys were afraid of pregnancy occurring at an early age.

*I would like to be older before getting pregnant, because then I can take better care of my child. (Female, Kalamu II)*

*I would rather that I am older before I give birth, because I must first study. If I get pregnant today, I will dishonor my parents. (Female, Bumbu)*

*I think it’s not good get pregnant when we are very young because we will have complications at the birth. (Female, Bumbu)*

*I have a child now, when I gave birth I lost all my friends, I have no time to go out and I take care of my child by myself. (Female, Kalamu II)*

*If you are young and you have a child but you live with your parents, it is a burden for them because they still have to take care of your child and yourself too. (Female, Bumbu)*

*I prefer to have a child later because if I have a child now, I will lose the chance to marry a good husband, the man I can love. (Female, Kalamu II)*

On the other hand, some adolescent girls who preferred to have an early pregnancy explained that:

*I would like to give birth very early because when the age advances, there are complications to bring [a baby] into the world. (Female, Bumbu)*

*The advantage of giving birth early is that even if I die very early, at least I will have left a child on the earth. (Female, Kalamu II)*

*I think that giving birth very early has an advantage. My big sister gave birth very early and now her child is 10 years old and they can grow up together. (Female, Kalamu II)*

*It is not good to delay the first pregnancy, because of menopause. In time it reached our mothers at the age of 45, but nowadays menopause could as early as 30 and 35, which is why girls prefer to give birth very early. (Female, Bumbu)*

For the adolescent boys, their fear of pregnancy stemmed from the financial implications, the reaction of their parents, the acquisition of a “bad” reputation in the neighborhood, and dishonor and shame pregnancy would bring to their family especially their parents. Some shared that pregnancy could interrupt their education and that of the girl.

*We’re afraid of impregnating someone’s daughter because we are young and do not have opportunities. This may be a burden for our parents. (Male, Kalamu II)*

*I’m really scared to make a girl pregnant. The news is going to spread everywhere and it will be a disgrace to my parents and myself. (Male, Bumbu)*

*In some families, as soon as you impregnate someone’s daughter pregnant, you have to compensate her and sometimes we can even go to jail. (Male, Kalamu II)*

In the case where a girl became pregnant, some of the adolescent boys shared that they would marry her.

*If I impregnate a girl, I must marry her because she’s the woman I love. (Male, Bumbu)*

*If a girl is pregnant with my child, I will only take the child. However, if she is well educated, then I will marry her. (Male, Kalamu II)*

If a woman had a prior pregnancy, majority of the adolescent boys would not marry her for a variety of reasons.

*I am afraid to marry a woman who has already given birth. I am afraid that the father of the child will want to have a relationship with the woman. (Male, Kalamu II)*

*I cannot marry a woman who has already given birth because children are unnecessary burdens, I will marry a woman who has not yet given birth. (Male, Bumbu)*

*I will marry a woman who has children only with me and not a woman who already has a child. (Male, Kalamu II)*

*It is not certain that my wife’s child will be grateful after I have raised him/her. (Male, Bumbu)*

*My parents always told me not to marry a woman who already has a child. (Male, Bumbu)*

#### Barriers to modern contraceptive use

In the urban population, both adolescent males and females experienced barriers that prevent them from accessing and using modern contraception. Both groups mentioned lack of information or misinformation of contraceptives, as well as community disapproval. Adolescent girls cited the judgmental attitudes of health providers; adolescent boys mentioned fear of side effects and stigma surrounding contraception use.

##### Lack of information and misinformation about contraceptive methods

Although both males and females had some knowledge about contraceptive methods, the FGD suggest that lack of knowledge and misconceptions about contraception are barriers to use. Adolescent girls seemed to have more knowledge of the different types of contraceptives than males; most could name at least one method, including condoms, pills, the Depo injection or the implant, in that order. However, they also cited pharmaceutical products, such as antimalarial (Quinine and Tetracycline) and deworming medications (Decaris, Vermox, Tanzol), which they believed to prevent pregnancy but do not. They also erroneously named traditional methods (calendar/rhythm and withdrawal) as modern.

*I know girls who use condoms to avoid getting pregnant and also to avoid certain diseases. There are also girls who take medication like decaris adult or child. (Female, Kalamu II)*

Virtually all the adolescent boys were familiar with the condom but were far less familiar with other modern methods. Some had knowledge about natural methods (withdrawal and the calendar method). Similar to adolescent girls, adolescent males incorrectly cited pharmaceutical products- Decaris, Tetracycline and Vermox- as modern methods. Additionally some males indicated other ways to avoid pregnancy: having the girl drink a lot of water or sit over a basin after sexual intercourse to get rid of the semen in her vagina.

*When I have sex without a condom, I drink a lot of water and the girl drinks a lot of water. We do not use drugs because medications can give complications and water does not. (Male, Bumbu)*

*I know a drug, Decaris, which a woman takes after sex. Once she urinates, there will be nothing left. (Male, Bumbu)*

Some males further explained that adolescents lacked information on where to access contraceptive methods.

*Some women do not use modern methods of contraception due to ignorance, because they have no idea where to go to get them. (Male, Kalamu II)*

##### Attitudes towards contraceptives

Both adolescent boys and girls had similar attitudes toward contraception. Overall, most girls felt that using a contraceptive method to prevent pregnancy was smart.

*I use contraceptive methods and my friends think I’m smart because many of them have children. (Female, Bumbu)*

*They will think we are smart, because the person who is not intelligent is the one who does not protect herself and can get pregnant. (Female, Bumbu)*

Most adolescent girls also felt that unmarried adolescent girls have the right to use contraceptives because they have sex and thus need to prevent early and unwanted pregnancy.

*All girls have the right to use contraceptive methods to protect themselves against diseases. (Female, Kalamu II)*

*We, girls, have the right to protect ourselves from getting pregnant. (Female, Bumbu)*

*A girl who is not yet married must use contraceptive methods to protect herself from early pregnancy because she also has sex like married people. (Female, Kalamu II)*

All adolescents in the urban FGD knew someone who had used a contraceptive method, citing the condom, pill, Depo injection and implant.

*I know a young woman in the neighborhood who uses the condom to avoid getting pregnant. (Female, Bumbu)*

*To avoid getting pregnant, I know a girl who uses depo provera. (Female, Kalamu II)*

*My big sister uses the depo provera injection. (Female, Kalamu II)*

By contrast, a minority of adolescent girls felt that only married women should use contraceptives.

*I think a girl who is not yet married cannot use a contraceptive method. (Female, Kalamu II)*

Regarding adolescent boys, the majority felt that contraceptive use was important to protect against early pregnancy and STI/HIV.

*Condoms are important and it’s good to use them because they protect against diseases and early pregnancies. (Male, Bumbu)*

*For me, I think the condom really protects against pregnancies, because prostitutes always use the condom and do not get pregnant. (Male, Kalamu II)*

*The condom protects against pregnancy because after sex, sperm remains in the plastic and does not go into the woman’s body, and the woman cannot get pregnant. (Male, Bumbu)*

Most of these adolescent boys indicated that their preferred method was the condom. But paradoxically, most expressed a negative attitude towards using condoms.

*People say that the condom reduces sexual pleasure. With some girls, as soon as you show her a condom, she reacts by saying that you suspect she has a disease. (Male, Kalamu II)*

*For me the condom does not protect completely because it sometimes tears during sex especially if it is not of good quality. (Male, Kalamu II)*

*Me, when I have sex with a girl with a condom, I do not feel pleasure that’s why I do not use condom. (Male, Bumbu)*

Some boys shared that their use of condoms was dependent on the nature of the sexual encounter.

*I use the condom only when I’m with another girl I do not know or a prostitute, but I do not use it when I’m with my girlfriend. (Male, Kalamu II)*

*I can meet a girl along the way and I am attracted. If I must have sex at this time, I am not going to use the condom because I did not plan to meet that girl that day (Male, Bumbu)*

Fear of side effects emerged as another reason for an adolescent’s nonuse of modern contraceptives.

*We are often told that the lubricant from the condom causes disease. Moreover even when you open the condom you smell a bad odor, it proves that the lubricant on the condom has microbes. (Male, Kalamu II)*

##### Community disapproval and stigma

Several adolescent girls shared that females experience shame and embarrassment when they attempt to purchase contraceptives from health centers or the pharmacy, due to community disapproval of adolescents’ participating in sexual activities.

*I think girls are ashamed go and get condoms in a health center. (Female, Bumbu)*

*Girls are sometimes ashamed of going to the pharmacy to buy the condom or the pill in front of everyone, people will think they have sex and that’s why they want to protect themselves. (Female, Kalamu II)*

One adolescent girl shared that at times she feels shame from even bringing up the topic of contraceptives to family members:

*We cannot talk to family members, they may think that I’m having sex, on the contrary I feel comfortable speaking with the big sisters [other girls or women] in the neighborhood (Female, Kalamu II)*

Similarly, adolescent boys experienced embarrassment and shame from buying condoms and going to the health centers.

*When we go to buy condoms in a pharmacy and meet an adult who came to buy medicine, we are ashamed to go inside and buy the condoms. (Male, Kalamu II)*

##### Perceptions of attitudes and knowledge of health providers

The anticipated reception from health providers was another reason for the nonuse of modern contraception and services at the health center. Many adolescent girls shared that personnel at pharmacies treated them poorly, at times were indiscrete and were reluctant to sell them modern contraceptives.

*I know of girls who have purchased contraceptives in pharmacies, but sometimes when you want to buy condoms, the pharmacist asks us to tell the person who sent us, they prefer to sell to adults. (Female, Bumbu)*

*For certain contraceptives, the pharmacist does not sell them, he says that in case of problems it is he that will be arrested. (Female, Kalamu II)*

Instead, adolescent girls preferred to visit health centers because they felt that the doctors and nurses were more knowledgeable and had a higher level of training on reproductive health services in comparison to pharmacists.

*I prefer the health center because I can meet with a nurse, but at the pharmacy that’s not the case, because pharmacists let other people sell in their place and these people do not know the drug. (Female, Bumbu)*

*At the health center, the doctors will examine you and then he will prescribe you medication while in the pharmacy you go directly to purchase drugs without the doctor’s prescription. (Female, Kalamu II)*

*I prefer the health center, because it gives information on how to protect against pregnancies and disease. (Female, Bumbu)*

Even though adolescent girls preferred health centers, they cited the unwillingness of many health personnel, including doctors and nurses, to provide modern contraception to adolescents.

*For us minors, it is difficult for nurses or doctors to give us contraceptive methods. (Female, Bumbu)*

##### Cost of modern contraception

A number of adolescent girls and boys cited cost of modern contraceptives and the inability to afford the services at the health center as barriers to their use. The prices of methods and services vary depending on the location of the service. Several adolescent females shared that:

*Some girls lack money to go to the health center. (Female, Kalamu II)*

*Other contraceptive methods are expensive and the price varies from US $10 to US $20, which is not within our reach. (Female, Kalamu II)*

Adolescent males shared the same sentiment; one participant suggested that condoms be given out for free.

*I think that women do not use these methods because they do not have the means to obtain them, they have no money. (Male, Bumbu)*

*For me, the condom must be free. Sometimes, I even don’t have 100 Fc ($0.10 U.S.). At the moment when I want to have sex with my girlfriends, I do it without a condom. (Male, Kalamu II)*

### Rural population

While adolescents from the urban areas were aged 15–19 years, the respondents from the rural areas were aged 15–24 years. Regardless of the age difference, common themes were identified in the urban and rural FGDs. Both discussed barriers to the use of contraceptive methods, perceptions of provider attitudes towards adolescents’ use of contraceptives and the social acceptability of the use contraception among adolescents. In addition, young people in the rural FGD expressed their views on their preferred family size and marriage, their motivation to engage in premarital sex and the social norms around premarital sex.

#### Desire for a marriage and pregnancy

The majority of the young women in the rural areas preferred to get married between the ages of 18 and 20 years.

*I must marry when I am about 20 years old because it is the age of maturity, and I can give birth without problems. At this age, I will be an adult and I will be able to manage my home well. (Female, Alungi)*

*I will get married after high school so that my future husband respects me. Also then I can go to university, find work and earn a living. (Female, Bili)*

*I want to get married at 19 because I want to give birth early. (Female, Bosobolo)*

The young men participating in the FGD shared that they preferred to get married between the ages of 20 and 25.

*I prefer to get married at 20 years old because first, I need to go to school and find a job before I get married in order to feed my family; I have to have money. (Male, Bosobolo)*

*We want to get married at married at 24 because we think we will be mature at this age. (Male, Mweka)*

A minority of young men and women wanted to get married early so they could have children early.

*I’m going to get married quickly because I am afraid of dying early and impregnating someone’s daughter. I want to be a young dad like my parents who were married at a young age. (Male, Alunguli)*

The majority of the young women wanted to have more than 6 children. Many who desired large families believed that with more children, they are more likely to have multiple sources of financial support in their old age; others felt it would help combat the fear of losing a child at a young age.

*I also want to have many children because among them, one can become a mason and build a house for us [parents], another can plow/cultivate a field, another can even go study and go abroad where he can properly support his family and our future will be assured. (Female, Mutoto)*

Males gave similar responses:

*I must give birth to more children, 8 or 9, because not all of them will live, there will also be those who die. (Male, Bosobolo)*

*I will have many children because one of the children may have lots of money and will help me in my old age. (Male, Alunguli)*

A minority of young women and men wanted fewer than six children because of financial and economic reasons:

*I will only have three children because I will be capable of providing for them, raising them because if you have many children, you need a lot of money too. (Female, Bili)*

*“I think I will have 4 children because here in Mweka it is not easy to find work, you must have few children to allow you to educate them and to feed them well” (Female, Mweka)*

*I will not have many children because life is expensive, it is necessary that we do not follow our parents, me I would like to take good care of my children. (Male, Mweka)*

*I will not have more than five children. If they are many, it will be difficult to finance their education, to feed them, to lodge and to clothe them. If I am not able to bear the costs, then they could find themselves in the street. (Male, Alunguli)*

#### Attitudes towards premarital sex

The majority of young men and women had a favorable opinion towards their peers who engaged in sexual activity before marriage for multiple reasons:

*For me having sex with boys before marriage is a proof that one is beautiful because the men come to you. (Female, Kampene)*

*We live together, if you do not have sex like the others, we will laugh at you and say that in marriage you will not be a good wife, that’s why we encourage sex before marriage. (Female, Mweka)*

*Girls have sex because they seek to practice before marriage, they do so out of curiosity and so that they are loved by the boy. (Female, Mutoto)*

*People think that if we do not have sex before marriage, we will not have children in the marriage and will have difficulty conceiving (Female, Mweka)*

Young women in the community may engage in sex for financial benefits and lack of parental supervision.

*Our parents are retired and they cannot do anything for us. The boy provides money, I know it comes at a cost, because he is not a family member (brother/father). When he asks to have sex, I will agree. (Female, Alunguli)*

*Some girls have sex because they imitate their colleagues who do it. When they see their girlfriends wear clothes that they bought with the money they got from boys, they will mimic them. (Female, Bili)*

*Because parents don’t monitor their children and the bad company that girls keep, they [girls] have sex before marriage (Female, Mutoto)*

Young men were also favorable to premarital sex - out of curiosity, peer pressure, a test of virility, a chance to gain experience, and their reward for giving a girl gifts.

*Me, I’m doing it [having sex] because I’m curious to know about the body of the girl and also to know if I am sexually active. (Male, Alunguli)*

*In our community, we are not waiting for marriage before having sex. When we see that our friends do it, we also begin to imitate that. (Male, Mweka)*

*A boy must have sex to test his manhood. His body must be in motion to be in good health. And girls respect us when we have sex. (Male, Bili)*

*We have sex because if we are not experienced, the woman can leave us because of sexual dissatisfaction. She will look for another man to satisfy her and so we must learn now so we don’t get a divorce one day. (Male, Kampene)*

*We sometimes have sex before marriage because we are told that if we do not have sex before we are 18, we will be impotent and people will mock us on our wedding day. (Male, Mutoto)*

*I have sex because girls ask for money and if they don’t have sex, then we waste/lose our money. (Male, Bosobolo)*

By contrast, a few young men and women had an unfavorable opinion of premarital sex. Their explanations focused on the health consequences, religious reasons and stigma surrounding premarital sex.

*If your friends learn that you have sex with boys, they will consider you a prostitute and you will feel shame. (Female, Bosobolo)*

*My friends tell me not to have sex before marriage because there are many diseases in the world. (Male, Bili)*

*I sometimes advise friends to finish their studies before having sex because they can jeopardize their future if the girl becomes pregnant, and then religion forbids sex. (Male, Bili)*

Whereas the majority of young people accepted premarital sex, they felt that adults in the community disapproved of sex.

*When the mothers of the village see us with boys, often they are not happy. They advise us to finish our studies and be careful about unwanted pregnancies. (Female, Bosobolo)*

*Adults are unhappy when they see us with boys and tell us that sex causes STIs and HIV. (Female, Mutoto)*

The young men indicated that adult disapproval of premarital sex stemmed from socio-cultural factors and risks associated with sex.

*Parents do not want their children to have sex before marriage, especially if their child is a girl. The custom here is that if the girl is a virgin when she gets married, the girl’s parents are given a gift, a goat. It is an honor for the parents and for the church where the girl prays. (Male, Mutoto)*

*Our parents often tell us this: we [the parents] do not understand why young people today want to have sex before marriage when in our time we did not do it. (Male, Bili)*

*Adults are not happy [if their children have sex] because parents have suffered a lot to raise their children. They expect to be helped by their children in their old age. With sex before marriage, young people are at risk of contracting diseases and compromising their future. (Male, Bosobolo)*

A few of the males cited cases where the adults in the community might condone sex before marriage:

*An adult in my village told me to have sex with the girls so that they would respect me. The old man said, if you do, you will be a strong and powerful man and the girls will respect you especially if it is satisfying. (Male, Alunguli)*

*For some irresponsible parents, when their daughter is friends with a boy who has money, they say nothing and they sometimes encourage them. (Male, Mweka)*

#### Barriers to modern contraceptive use

Young people in the rural population experienced barriers to contraceptive use similar to those found in Kinshasa: lack of knowledge and misinformation of contraceptive methods, negative attitudes towards contraceptives, and judgmental attitudes among health providers.

##### Lack of knowledge and misinformation

The young women and men in the rural FGD were knowledgeable about some forms of contraception. Young women tended to know at least one method, including condoms, the calendar method, Depo injection, implants, the pill, abstinence, and withdrawal. Young women did not express a preference for any one method over another: their choice would depend on the situation. Young people incorrectly cited some pharmaceutical products, such as Decaris, Paracetamol, Tanzol Ampicillin as contraceptives. A few sought advice from parents, doctors, nurses and friends.

*Me, when I need to avoid pregnancy, I go to my sister’s house in the neighborhood who tells me this: just take 2 or 3 paracetamol tablets before intercourse and you will not get pregnant. (Female, Bosobolo)*

*Often I go to my mother who teaches me how to calculate my dates [calendar method]. (Female, Kampene)*

The methods most commonly mentioned by the young men were condoms, rhythm, withdrawal and abstinence.

*I am positive that my friends have sex. I just advise them to wear a condom to prevent pregnancy or disease. (Male, Mweka)*

*The most appropriate method for us boys is the condom. This method is good especially for girls who have an irregular cycle because we do not know their cycle. (Male, Bili)*

*To avoid pregnancy, we must also follow the dates of the girls because during the month, the woman has a bad [fertile] period. (Male, Bosobolo)*

*There is a method of throwing semen on the ground instead of directly in the female organ [vagina]. (Male, Mutoto)*

For information or advice, young men would consult health providers at health centers, their parents or teachers.

*When we need information, we will go in the health facilities to speak with the health staff. At school, we must consult the teachers. They are experts, they tell us how to protect ourselves against diseases. (Male, Kampene)*

*I ask my family members because they have had children before and they have a lot of information on how to avoid sex. (Male, Bili)*

*When I need information on how to avoid a pregnancy, I go to a health facility with doctors or nurses. They advise us to use the condom during sex to avoid pregnancy. (Male, Bili)*

##### Attitudes towards contraceptive methods

Male attitudes were a major barrier to use. Most young men agreed that it was important to use contraceptives but felt it was the girl’s responsibility to prevent pregnancy.

*It’s the girl who is responsible for the contraception. She knows her monthly cycle and she must say when she cannot have sex with a man because she is in her fertile period. (Male, Mweka)*

*It is the girl who is responsible because she can refuse the gifts given to her by the boy, also she knows her menstral cycle. (Male, Kampene)*

A few males who thought it was also their own responsibility:

*The boy is responsible because he asked the girl to have sex. He must take every precaution to avoid pregnancy. (Male, Bosobolo)*

##### Perceptions of provider attitudes towards youths’ contraceptive use

Majority of the young women felt that the health providers had a negative attitude towards their use of contraception, which discouraged them from using services:

*At the hospital, I was getting the Depo Provera injection. The doctor asked me if I had given birth before: I told him no and he forbade me to continue taking it. He said it is forbidden to girls who have not yet given birth, because it makes them sterile. He told me to keep track of my dates (calendar method). (Female, Bosobolo)*

*The nurses do not receive us well because when we ask questions, they see us as bad girls who seek prostitution, thus I cannot ask the nurses or the doctors. (Female, Mweka)*

Conversely, young males had mixed experiences with providers:

*The providers receive us well at the health center, they like people who come asking for advice on preventing pregnancy. (Male, Mweka)*

*One day I went to the health center to ask for information about sex, a nurse who scolded me. And so, I will not be returning over there for fear of being scolded again. (Male, Alunguli)*

## Discussion

The bustling city of Kinshasa with over 11 million people and the relatively isolated rural areas of the DRC seem worlds apart, yet the SRH problems facing young people are surprisingly similar. Despite differences in age of the participants and specific topics covered, this research points to common experiences: premarital sex largely sanctioned by peers but not adults, fear of pregnancy, limited knowledge of contraceptive methods, misinformation that certain common pharmaceutical products (e.g., decaris) prevent pregnancy, barriers to accessing contraceptives from health facilities and pharmacies, shame and stigma.

A few differences did emerge. Urban participants cited cost as a barrier to contraception, whereas rural participants did not. Contraceptives are provided free of charge in rural health zones supported by ASSP. Similarly, only female participants in the urban areas mentioned the judgmental attitude of health personal as a barrier to contraception use, whereas both males and females in rural areas did. Rural participants perceived contraceptive use as a female’s domain, whereas urban participants did not discuss this topic.

We acknowledge several limitations of the research. The FGD guides covered slightly different topics and the focus group participants differed in age in the urban and rural areas. Findings from FGD cannot be generalized to the larger population. The selection of “strong” versus “weak” health zones based on FP performance used to select the communities in the larger study may have limited relevance to SRH among young people. The process of transcribing audiotapes from the local language in French, then producing quotations in English may distort the original meaning.

Our findings are consistent with other studies from the DRC. The 2013–2014 DHS showed that men and women want large families of 7 and 6 children respectively [1]. The DHS study also demonstrates that awareness of contraception is high [1]. The recent PMA2020 report for Kinshasa shows a gap between the average onset of sexual activity (17.3 years), and the first use of contraception (20.5 years) [24]. The DHS reported that 27% of 15–19 years old women have begun child bearing [1]. Another quantitative survey showed that the majority (54%) of unmarried sexually active youth did not use contraception because they were not married [25]. A qualitative study in the DRC among women aged 15–35 years revealed that women frequently used deworming or anti-malarial medicines after sex as a form of emergency contraception [26]. Similar to young people in our study, qualitative research from Eastern DRC among adults yielded highly similar results to the current study on SRH themes [27]. Adults also shared similar concerns and experiences related to shame and stigma associated with using contraception [12, 13, 27].

Our findings are also similar to SRH results reported elsewhere in sub-Saharan Africa. A study in Zimbabwe found that adolescents feared pregnancy and its potential social, financial, educational and health impact [28]. A systematic review conducted to explore the limits of modern contraceptive use found that lack of knowledge hindered use [29]. In Ethiopia, a study found that among health workers with an unfavorable attitude towards providing FP to adolescents, some indicated setting up penal rules and regulations against adolescents that practice premarital sex [30]. Attitudes of health care providers discouraged young men and women from seeking care [30–32]. Similar to our findings, stigma and shame discouraged Tanzanian adolescents from seeking reproductive health care [33].

Prior to 2015, SRH programming in the DRC was very limited, even in Kinshasa. Only 20 of 516 health zones nationwide offered any type of SRH service for young people. As of 2017, however, 120 health zones have funding for SRH programming [20]. The PNSA coordinated the development of a National Strategic Plan for the Health and Wellbeing of Adolescents and Youth 2016–2020, which provides a vision for future programming and has attracted external donor funding to this largely neglected area [34]. As shown in this study, the needs for information and services are great in both urban and rural areas. Yet in a country with widespread resistance only a few years ago, SRH programming is gaining increasing acceptance among political decision-makers, community opinion leaders, and other gatekeepers.

## Conclusion

Although the DRC has achieved gains in acceptance in SRH programming, our findings signal a pressing need for information and services for young people in both urban and rural areas. The widespread acceptance of premarital sex among adolescents, the limited knowledge of contraception methods, and misinformation that certain pharmaceutical products prevent pregnancy increase their risk for pregnancy and STIs. The findings also suggest that fear of pregnancy, the judgmental attitude of health providers, and fear of side effects are major concerns among adolescents and young people within the urban and rural areas. Addressing the SRH needs of adolescents and young people can have life-long protective benefits. As DRC continues to expand its implementation of SRH programming, it is vital to address the persistent needs and challenges of adolescents and young people for access to modern contraception and other SRH services.

## Abbreviations

ASSP: Accès au Soins de Santé Primaire
DHS: Demographic and Health Survey
DRC: Democratic Republic of Congo
FGD: Focus group discussion
FP: Family planning
HIV: Human immunodeficiency virus
PMA2020: Performance Monitoring and Accountability 2020
PNSA: Programme National de Santé de L’Adolescent (National Program of Youth and Adolescent Health)
SRH: Sexual and reproductive health
STI: Sexually transmitted infections
